# Depression, antidepressant medications, and risk of *Clostridium difficile* infection

**DOI:** 10.1186/1741-7015-11-121

**Published:** 2013-05-07

**Authors:** Mary A M Rogers, M Todd Greene, Vincent B Young, Sanjay Saint, Kenneth M Langa, John Y Kao, David M Aronoff

**Affiliations:** 1 Department of Internal Medicine, University of Michigan, 016-440E NCRC, Ann Arbor, MI 48109-2800, USA; 2 Ann Arbor Veterans Affairs Medical Center, Ann Arbor, MI, USA

**Keywords:** *Clostridium difficile*, Colitis, Depression, Antidepressant medication

## Abstract

**Background:**

An ancillary finding in previous research has suggested that the use of antidepressant medications increases the risk of developing *Clostridium difficile* infection (CDI). Our objective was to evaluate whether depression or the use of anti-depressants altered the risk of developing CDI, using two distinct datasets and study designs.

**Methods:**

In Study 1, we conducted a longitudinal investigation of a nationally representative sample of older Americans (n = 16,781), linking data from biennial interviews to physician and emergency department visits, stays in hospital and skilled nursing facilities, home health visits, and other outpatient visits. In Study 2, we completed a clinical investigation of hospitalized adults who were tested for *C. difficile* (n = 4047), with cases testing positive and controls testing negative. Antidepressant medication use prior to testing was ascertained.

**Results:**

The population-based rate of CDI in older Americans was 282.9/100,000 person-years (95% confidence interval (CI)) 226.3 to 339.5) for individuals with depression and 197.1/100,000 person-years for those without depression (95% CI 168.0 to 226.1). The odds of CDI were 36% greater in persons with major depression (95% CI 1.06 to 1.74), 35% greater in individuals with depressive disorders (95% CI 1.05 to 1.73), 54% greater in those who were widowed (95% CI 1.21 to 1.95), and 25% lower in adults who did not live alone (95% CI 0.62 to 0.92). Self-reports of feeling sad or having emotional, nervous or psychiatric problems at baseline were also associated with the later development of CDI. Use of certain antidepressant medications during hospitalization was associated with altered risk of CDI.

**Conclusions:**

Adults with depression and who take specific anti-depressants seem to be more likely to develop CDI. Older adults who are widowed or who live alone are also at greater risk of CDI.

## Background

*Clostridium difficile* infection (CDI) is the most commonly diagnosed cause of antibiotic-associated diarrhea, and has emerged as a major nosocomial infection, surpassing methicillin-resistant *Staphylococcus aureus* in some hospitals [1]. In addition to causing more than 7,000 deaths annually in the USA [2], it is prevalent in hospitals throughout Europe, with a mean frequency of 23 cases for every 10,000 admissions [3]. Concerted efforts are ongoing to identify modifiable risk factors for CDI, motivated by the need for better preventive and therapeutic options against this infection.

Modifiable risk factors for CDI include several classes of medications such as proton-pump inhibitors (PPIs) and histamine-2 receptor antagonists (H2RAs) [4]. Anti-depressants have also been implicated in this infection [5]. In a cohort study of 14,719 hospitalized patients, an ancillary finding of an association between antidepressant medications and CDI was reported, which was stronger than that found for PPIs or H2RAs [5]. A possible link between antidepressant medications and CDI is noteworthy because depression is the third most prevalent disabling condition worldwide, as reported by the World Health Organization [6]. Effects of depression have been shown to include alterations in the gut microbiota and increased intestinal permeability [7-9]. In the murine model, behavioral depression increases jejunal permeability and susceptibility to colitis [10]. In population-based human studies, depression was found to be significantly predictive of inflammatory bowel disease [11,12].

Since the publication of the study by Dalton *et al*. linking antidepressant use with the risk of CDI [5], there has been no evidence, to our knowledge, to support or refute this finding. Therefore, we planned an investigation to assess whether depression or the use of anti-depressants alters the risk of CDI. We tested these hypotheses using two distinct but complementary approaches. First, we assembled longitudinal data from a nationally representative sample of older Americans so that these relationships could be assessed in a more generalizable population. Second, we utilized a clinical database of adults admitted to an academic hospital to obtain details regarding antidepressant use.

## Methods

### Ethics approval

The studies received human subjects approval at the University of Michigan. Patient consent was obtained for subjects who participated in the Health and Retirement Study. Because retrospective electronic data were used for the clinical study, patient consent was waived.

### Study 1

We conducted a longitudinal study to determine population rates of CDI in individuals with and without depression, and to evaluate the association between depression and CDI. Subjects were participants in the nationally representative Health and Retirement Study (HRS), which is an ongoing longitudinal study of older Americans. Details of the study design of the HRS and subject characteristics have been published previously [13]. Households were selected using multistage area probability sampling from the National Sample frame, with random selection of eligible individuals within the household. Subjects were interviewed every 2 years; data from interviews during years 1992–2006 were used in the present investigation. Data from the interviews were linked to files from the Centers for Medicare and Medicaid Services (CMS) covering the period 1991–2007, for fee-for-service beneficiaries. The CMS Standard Analytical Files used were the Inpatient, Outpatient, Skilled Nursing Facility, Carrier (Part B), Home Health Agency, and Denominator files.

CDI was based on diagnosis by a physician of CDI (ICD-9-CM code 008.45) recorded in Inpatient, Outpatient, Carrier, Skilled Nursing Facility or Home Health Agency files. This captured both outpatient and inpatient diagnoses of CDI. The positive and negative predictive values of the ICD-9-CM code for identifying CDI have been previously reported as 87% and 96%, respectively [14]. Depression was determined from a physician diagnosis of major depression (ICD-9-CM codes 296.2×, 296.3×, 300.4, 311) at any time prior to diagnosis of CDI. We also captured a wider group of patients with ‘depressive disorders’ which included brief or prolonged depressive reactions, adjustment reaction with anxiety and depression, depressive type psychosis, chronic depressive personality disorder, bipolar affective disorder with depression, neurotic depression, depressive disorder (not elsewhere classified), and major depression (ICD-9-CM codes 296.2×, 296.3×, 296.5, 298.0, 300.4, 301.12, 309.0, 309.1, 309.28, 311, V79.0) diagnosed at any time prior to the diagnosis of CDI. Psychiatrist visits were determined from the Carrier (individual provider) file using Berenson-Eggers Type of Service (BETOS) code M5B (evaluation and management by a specialist – psychiatry). Other items related to depression were determined from the initial interview of the HRS. The questions were: ‘During the past week, did you feel sad?’ and ‘Did a doctor ever tell you that you had emotional, nervous or psychiatric problems?’ We also assessed marital status at the time of the first interview (married or partnered, divorced or separated, single, widowed). As household information was available, we also extracted data regarding the number of people in the household at the time of the first interview.

Information was also extracted from the HRS for year of birth, gender, race, ethnicity, region of residence (northeast, midwest, south, west), body mass index (BMI; kg/m^2^) at the time of the first interview, smoking status at the time of the first interview (never, former, current), end-stage renal disease at the time of the first interview, and diagnoses of Crohn’s disease, irritable bowel syndrome, ulcerative colitis, or celiac disease (prior to the first CDI diagnosis for those with CDI). We anticipated that some individuals would have greater contact with physicians and medical services and, therefore, we also calculated the number of infection-related visits (inpatient and outpatient) and the total number of medical-related visits (inpatient and outpatient) over the entire period of observation for each subject. The number of infection-related visits was the crude proxy for exposure to antibiotics. Missing values were imputed for the variables of race (n = 37), ethnicity (n = 3), region of residence (n = 28), BMI (n = 30) and smoking status (n = 91). The calendar year of the first interview was also included to account for secular trends.

Survey-weighted population-based rates of CDI (number of individuals with the diagnosis of CDI per 100,000 person-years of observation) were calculated for individuals with and without major depression. The rates were stratified by subject characteristics (age, gender, race, ethnicity, BMI, education and region of residence). Survey-weighted logistic regression (svy: logistic) was used to assess the association between depression and CDI, offset by the natural log of the person-years under observation, after adjustment for year of birth (centered), gender, race, ethnicity, BMI, smoking, end-stage renal disease, Crohn’s disease, celiac disease, ulcerative colitis, irritable bowel disease, number of infection-related visits, and total number of medical-related visits. Alpha was set at 0.05, two-tailed.

### Study 2

A hospital-based case–control study was conducted to evaluate the association between antidepressant medications and hospital-acquired CDI. Subjects were all adult patients (18 years or older) hospitalized in the University of Michigan Health System (UMHS) during the period August 2010 to February 2012, who had their stools tested for *C. difficile*. Stool was tested for *C. difficile* by the UMHS Clinical Microbiology Laboratory. Testing was performed on stools for glutamate dehydrogenase (GDH) antigen and toxins A or B (C. DIFF QUIK CHEK COMPLETE^®^ test; Techlab, Inc., Blacksburg, VA, USA). All antigen/toxin discordant stool tests were subjected to analysis for the tcdB gene by real-time PCR (BD GeneOhm™ Cdiff Assay; Franklin Lakes, NJ, USA). Because hospital-acquired infection was of interest, patients admitted for reason of *C. difficile* (principal diagnosis of CDI) were excluded. Cases were patients who tested positive for CDI at 48 hours or longer after admission and controls were patients who tested negative for CDI at 48 hours or longer after admission. Because CDI testing is usually ordered for symptomatic patients (for example, those with antibiotic-associated diarrhea), cases and controls were suspected *a priori* to be fairly concordant on these general characteristics. For individuals with multiple *C. difficile* tests, the first test result was used for the purposes of this study. Data regarding patient demographics, comorbidities and medications (prior to the date of stool collection for CDI testing) were extracted from the electronic hospital data system. Detailed information was available for the dosages and dates in which medications were given to each patient.

To assess differences in patient characteristics by case–control status, Pearson’s χ^2^ test for categorical data, Student’s *t*-test for differences in mean age, and the Kruskal-Wallis test for differences in length of stay prior to stool collection were used. Unconditional logistic regression was used to assess the association between antidepressant use and CDI, with and without adjustment for age, gender, race, antibacterial medications, PPIs, H2RAs, statins, irritable bowel syndrome, celiac disease, Crohn’s disease and ulcerative colitis.

Finally, we conducted a sensitivity analysis for the case–control study using different controls. We conducted a case-crossover study in which each patient served as his/her own control, to evaluate differences in anti-depressants while controlling for any history of depression. This analysis incorporates a within-person comparison in which the use of medications is compared over different time periods for the same patient and therefore, it can be used to separate the effects of the anti-depressants from the effects of the disease itself (pathophysiology of depression). Such an approach also controls for factors that are difficult to capture, such as genetic profile and past dietary habits. UMHS hospitalizations in which hospital-acquired CDI occurred (n = 406) were compared with subsequent hospitalizations (n = 949) for the same patient in which CDI did not occur (July 2009 to June 2012). Odds ratios (ORs) were calculated using a conditional logit model for panel data (clogit), offset by the natural log of the time at risk for infection (length of stay for hospitalizations without CDI; length of time from admission to stool collection for positive *C. difficile* for hospitalizations with CDI). Statistical analyses were performed by using Stata/MP 11.2 (StataCorp LP, College Station, TX, USA). Alpha was set at 0.05, two -tailed.

## Results

### Study 1

The age (mean ± SD) of the 16,781 participants in the linked HRS-CMS database at the time of the first interview was 67.9 ± 10.6 years and 56.2% were women (Table 1). At baseline, the majority were either overweight (BMI 25.0 to 29.9 kg/m^2^) or obese (BMI ≥30 kg/m^2^). Of the 16,781 subjects, 404 had been diagnosed with CDI at least once, according to their Medicare records. Of these 404 people diagnosed with CDI, 142 (35%) had received a diagnosis of major depression and 150 (37%) had received a diagnosis of a depressive disorder prior to CDI. The rates of CDI in the reference population (fee-for-service Medicare population in the USA) are given in Table 1, stratified by major depression. The rate of CDI was 282.9 per 100,000 person-years in those with depression, and 197.1 per 100,000 person-years in those without depression.

**Table 1:** **Rate of *Clostridium difficile *infection by previous diagnosis of major depression in Medicare beneficiaries**

**Characteristics**	**Sample, n (%)**	**No depression**		**Depression**	
		**Rate**^**a**^	**95% CI**	**Rate**^**a**^	**95% CI**
Age at first interview^**b**^					
<60 years	5277 (31.4)	125.1	82.2 to 168.0	332.9	193.5 to 472.2
60 to 75 years	7258 (43.3)	178.2	145.2 to 211.1	239.8	182.9 to 296.7
>75 years	4246 (25.3)	301.0	241.6 to 360.3	321.6	213.4 to 429.9
Gender					
Men	7352 (43.8)	179.6	145.5 to 213.6	268.9	163.5 to 374.3
Women	9429 (56.2)	212.2	173.6 to 250.8	289.3	233.6 to 345.1
Race					
African-American	2232 (13.3)	221.5	136.7 to 306.2	346.4	173.6 to 519.3
Other	14549 (86.7)	193.1	161.7 to 224.5	275.1	214.5 to 335.7
Ethnicity					
Mexican-American	700 (4.2)	243.7	77.3 to 410.1	313.1	65.6 to 560.7
Other Hispanic	486 (2.9)	204.9	272.4 to 382.6	234.2	129.8 to 338.6
Non-Hispanic	15595 (92.9)	195.1	165.5 to 224.6	283.6	222.3 to 344.9
Body mass index					
<18.5 kg/m^2^	366 (2.2)	348.2	87.9 to 608.6	475.5	115.3 to 835.6
18.5 to 24.9 kg/m^2^	6311 (37.6)	185.0	149.2 to 220.8	279.0	185.7 to 372.2
25.0 to 29.9 kg/m^2^	6851 (40.8)	188.4	151.7 to 225.1	300.8	210.7 to 390.9
≥30.0 kg/m^2^	3253 (19.4)	225.4	160.6 to 290.1	234.9	133.5 to 336.3
Education					
No high-school degree	5691 (33.9)	247.6	203.2 to 291.9	254.4	170.5 to 338.2
High-school degree	11090 (66.1)	172.1	137.7 to 206.5	299.6	234.0 to 365.3
Region of residence					
Northeast	2958 (17.6)	265.3	193.1 to 337.4	366.5	194.9 to 538.1
Midwest	4205 (25.1)	226.6	155.5 to 297.8	307.0	191.5 to 422.5
South	6823 (40.7)	176.5	135.4 to 217.7	257.5	185.6 to 329.5
West	2795 (16.7)	136.1	85.4 to 186.7	218.8	115.6 to 322.0
Overall	16781	197.1	168.0 to 226.1	282.9	226.3 to 339.5

^a^Number of individuals with CDI/100,000 person-years.

^b^Age range 36 to 103 years.

After adjusting for demographic characteristics, comorbidities and frequency of medical visits, there was a 36% increase in the odds of developing CDI for individuals with major depression compared with those without major depression (Table 2). The findings were similar for depressive disorders (OR = 1.35), feelings of sadness (OR = 1.41) and emotional, nervous or psychiatric problems (OR = 1.47). Older Americans who were widowed were significantly more likely to develop CDI; the odds of CDI were 54% greater in older adults who were widowed compared with those who were married. Being divorced or separated was significantly associated with CDI in the unadjusted model but not in the fully adjusted model (*P* = 0.072). Developing CDI was also more common in older adults who lived alone; individuals who lived with others had a 25% decrease in the odds of developing CDI compared with those who lived alone.

**Table 2:** **Odds ratios for *Clostridium difficile *infection and previous depression-related conditions in Medicare beneficiaries**

	**Unadjusted**			**Adjusted for age, gender, race**			**Fully adjusted**^**a**^		
**Conditions**	**OR**	**95% CI**	***P *value**	**OR**	**95% CI**	***P *value**	**OR**	**95% CI**	***P *value**
Depression									
Yes	1.45	1.14 to 1.86	0.003	1.41	1.10 to 1.82	0.008	1.36	1.06 to 1.74	0.016
No	1.00	(reference)		1.00	(reference)		1.00	(reference)	
Depressive disorders									
Yes	1.44	1.13 to 1.85	0.004	1.40	1.08 to 1.80	0.011	1.35	1.05 to 1.73	0.021
No	1.00	(reference)		1.00	(reference)		1.00	(reference)	
Feeling sad									
Yes	1.62	1.27 to 2.06	<0.001	1.48	1.16 to 1.90	0.002	1.41	1.10 to 1.81	0.008
No	1.00	(reference)		1.00	(reference)		1.00	(reference)	
Emotional, nervous or psychiatric problems									
Yes	1.45	1.02 to 2.05	0.038	1.55	1.08 to 2.21	0.017	1.47	1.02 to 2.11	0.041
No	1.00	(reference)		1.00	(reference)		1.00	(reference)	
Psychiatrist visit									
Yes	1.42	1.10 to 1.83	0.008	1.35	1.05 to 1.72	0.020	1.26	0.95 to 1.67	0.104
No	1.00	(reference)		1.00	(reference)		1.00	(reference)	
Marital status									
Widowed	1.84	1.49 to 2.28	<0.001	1.59	1.26 to 2.02	<0.001	1.54	1.21 to 1.95	0.001
Divorced/separated	1.63	1.02 to 2.58	0.040	1.66	1.02 to 2.70	0.043	1.54	0.96 to 2.45	0.072
Single	1.29	0.63 to 2.65	0.473	1.27	0.61 to 2.63	0.518	1.28	0.62 to 2.66	0.499
Married	1.00	(reference)		1.00	(reference)		1.00	(reference)	
Multiple people in household									
Yes	0.67	0.55 to 0.81	<0.001	0.71	0.58 to 0.88	0.002	0.75	0.62 to 0.92	0.007
No	1.00	(reference)		1.00	(reference)		1.00	(reference)	

^a^Adjusted for year of birth (centered), gender, race, ethnicity, BMI, smoking, end-stage renal disease, Crohn’s disease, celiac disease, ulcerative colitis, irritable bowel disease, number of infection-related visits, total number of medical-related visits, and calendar year of first interview.

### Study 2

In Study 2, there were 4,047 adult patients who had their stools tested for *C. difficile* during hospitalization within the UMHS from 1 August 2010 to 29 February 2012. Of these subjects, 468 tested positive (cases) and 3579 tested negative (controls). The characteristics of the patients are given in Table 3 and show that the cases and controls were similar with regard to age, gender, race, type of admission, number of days from admission to the time of stool collection for testing, and use of various medications prior to stool collection. As seen in Table 3, 83.8% of the cases and 82.6% of the controls had received antibiotics (*P* = 0.530). We also had data for both antibiotics and anti-depressants. In this study, 82.8% of those receiving anti-depressants were given antibiotics, and 82.7% of those not receiving anti-depressants were given antibiotics (*P* = 0.957).

**Table 3:** **Characteristics of hospitalized adults who tested positive (cases) and negative (controls) for *Clostridium difficile***

**Characteristics**	**Cases, n = 468**	**Controls, n = 3579**	***P *value**
Age, mean ± SD^a^	57.6 ± 17.5	58.8 ± 16.5	0.158
Female, n (%)	223 (47.6)	1723 (48.1)	0.841
Caucasian, n (%)	387 (82.7)	2927 (81.8)	
African-American, n (%)	51 (10.9)	386 (10.8)	
Other race/unknown, n (%)	30 (6.4)	266 (7.4)	0.727
Elective admission, n (%)	138 (29.5)	930 (26.0)	
Urgent admission, n (%)	104 (22.2)	846 (23.6)	
Emergent admission, n (%)	226 (48.3)	1803 (50.4)	0.268
Days until stool collection, median (IQR)^b^	6 (4 to 10)	6 (4 to 10)	0.273
Antibiotics, n (%)	392 (83.8)	2956 (82.6)	0.530
Immunosuppressants, n (%)	71 (36.4)	554 (35.0)	0.692
Proton-pump inhibitors, n (%)	268 (57.3)	2040 (57.0)	0.913
Histamine-2 receptor antagonists, n (%)	138 (29.5)	1061 (29.6)	0.944
Statins, n (%)	130 (27.8)	927 (25.9)	0.385
Anxiolytics, n (%)	67 (34.4)	499 (31.5)	0.419
Antipsychotics, n (%)	24 (12.3)	202 (12.7)	0.860

^a^Age range 18 to 100 years.

^b^Number of days from admission to stool collection for *Clostridium difficile* testing, median and interquartile range (IQR).

Certain anti-depressants were significantly related to CDI (Table 4). The odds of testing positive for *C. difficile* were twice as high in patients who received mirtazapine than in those who did not (OR = 2.14). For each dose of mirtazapine given, the odds of testing positive for *C. difficile* increased by 8%. There was also an association between fluoxetine and testing positive for *C. difficile,* with an OR of 1.92 after adjustment. For each dose of fluoxetine received, the odds of testing positive increased by 6%. Other selective serotonin reuptake inhibitors (SSRIs), besides fluoxetine, were not significantly associated with a positive CDI test. Of the tricyclic anti-depressants, each dose of nortriptyline was associated with an 11% increase in the odds of testing positive for *C. difficile*, although the OR for use (yes/no) was not significant at the 0.05 alpha level (*P* = 0.062). The remaining anti-depressants were not associated with a positive *C. difficile* test.

**Table 4:** **Odds ratios for antidepressant medications and *Clostridium difficile *infection in hospitalized adults**

		**Unadjusted**			**Adjusted**^**a**^		
**Medications**	**Using medication, n**	**OR**	**95% CI**	***P *value**	**OR**	**95% CI**	***P *value**
Noradrenergic and specific serotonergic anti-depressants							
Mirtazapine							
Use (versus no use)	99	2.11	1.29 to 3.45	0.003	2.14	1.30 to 3.52	0.003
Doses given, n		1.08	1.01 to 1.16	0.018	1.08	1.01 to 1.16	0.020
Selective serotonin reuptake inhibitors							
Fluoxetine							
Use (versus no use)	99	1.98	1.20 to 3.26	0.008	1.92	1.16 to 3.17	0.012
Doses given, n		1.06	1.00 to 1.12	0.036	1.06	1.00 to 1.12	0.046
Escitalopram							
Use (versus no use)	80	0.97	0.48 to 1.95	0.929	0.98	0.48 to 1.97	0.945
Doses given, n		1.04	0.98 to 1.10	0.191	1.04	0.98 to 1.09	0.234
Citalopram							
Use (versus no use)	310	0.90	0.62 to 1.32	0.599	0.90	0.62 to 1.31	0.569
Doses given, n		0.99	0.94 to 1.04	0.620	0.98	0.94 to 1.04	0.566
Sertraline							
Use (versus no use)	207	0.90	0.58 to 1.42	0.666	0.88	0.56 to 1.39	0.583
Doses given, n		0.98	0.93 to 1.04	0.486	0.98	0.92 to 1.04	0.461
Paroxetine							
Use (versus no use)	78	0.87	0.42 to 1.82	0.716	0.86	0.41 to 1.80	0.692
Doses given, n		0.98	0.88 to 1.09	0.693	0.98	0.88 to 1.09	0.680
Tricyclic anti-depressants							
Nortriptyline							
Use (versus no use)	49	1.98	0.98 to 4.00	0.056	1.96	0.97 to 3.97	0.062
Doses given, n		1.11	1.02 to 1.20	0.015	1.11	1.02 to 1.20	0.017
Amitriptyline							
Use (versus no use)	63	1.45	0.73 to 2.88	0.284	1.43	0.72 to 2.84	0.307
Doses given, n		1.04	0.96 to 1.14	0.337	1.04	0.96 to 1.14	0.343
Serotonin antagonist and reuptake inhibitors							
Trazodone							
Use (versus no use)	405	1.23	0.91 to 1.66	0.182	1.21	0.90 to 1.65	0.211
Doses given, n		1.04	0.98 to 1.10	0.184	1.04	0.98 to 1.10	0.197
Serotonin-norepinephrine reuptake inhibitors							
Duloxetine							
Use (versus no use)	82	1.19	0.63 to 2.26	0.597	1.15	0.60 to 2.19	0.681
Doses given, n		1.01	0.94 to 1.09	0.690	1.01	0.94 to 1.09	0.788
Venlafaxine							
Use (versus no use)	58	0.72	0.29 to 1.81	0.482	0.71	0.28 to 1.78	0.464
Doses given, n		1.05	0.95 to 1.16	0.367	1.05	0.94 to 1.16	0.401
Norepinephrine-dopamine reuptake inhibitors							
Bupropion							
Use (versus no use)	97	1.08	0.59 to 2.00	0.801	1.02	0.55 to 1.89	0.948
Doses given, n		0.95	0.85 to 1.06	0.357	0.94	0.85 to 1.05	0.306

a. Adjusted for age, gender, race, antibacterial medications, PPIs, H2RAs, statins, irritable bowel syndrome, celiac disease, Crohn’s disease, and ulcerative colitis.

In a secondary analysis, we considered whether interactions between anti-depressants could alter the risk of developing CDI. There was a significant interaction between mirtazapine and trazodone (*P* = 0.040 for the interaction term). For a patient receiving both of these anti-depressants, the odds of a positive *C. difficile* test were 5.72 times greater than in individuals receiving neither of these drugs (95% CI 2.01, 16.26; *P* = 0.001). Of the patients receiving mirtazapine only, 21.2% had a positive *C. difficile* test, whereas of the patients receiving trazodone only, fewer (13.6%) had a positive test. Of the patients receiving both mirtazapine and trazodone, 43.7% subsequently had a positive *C. difficile* test.

In a sensitivity analysis using a case-crossover design in which patients (n = 406) served as their own controls, the interaction between mirtazapine and trazodone remained significant (*P* = 0.010) after adjustment for antibacterial medications, statins, immunosuppressant medications, red blood cell transfusions, PPIs, and H2RAs (Table 5). The odds of CDI were greater during hospitalizations in which the patients received mirtazapine with trazodone compared with hospitalizations in which these same patients did not receive these two anti-depressants. However, the attributable risk associated with these two drugs was small, because the percentage of hospitalizations in which patients received both of these anti-depressants was only 1.25%.

**Table 5:** **Odds ratios for antidepressant medications and *Clostridium difficile *infection; case-crossover study of hospitalized adults**

**Medication**	**Hospitalizations, n**^**a**^	**OR**^**b**^	**95% CI**	***P *value**
Fluoxetine	53	1.74	0.46 to 6.65	0.416
Escitalopram	25	0.57	0.12 to 2.79	0.491
Citalopram	128	0.41	0.17 to 0.99	0.047
Sertraline	105	0.58	0.21 to 1.57	0.282
Paroxetine	18	4.71	0.88 to 25.33	0.071
Nortriptyline	30	0.22	0.02 to 2.13	0.190
Amitriptyline	38	1.20	0.30 to 4.79	0.800
Duloxetine	37	0.44	0.10 to 1.89	0.267
Venlafaxine	27	5.05	0.58 to 44.15	0.143
Bupropion	36	0.48	0.11 to 2.05	0.323
Mirtazapine	64	0.82	0.31 to 2.13	0.682
Trazodone	171	0.55	0.33 to 0.90	0.018
Mirtazapine and trazodone^c^	17	32.54	2.29 to 462.9	0.010

^a^Number of hospitalizations in which medication was administered.

^b^Adjusted for antibacterial medications, PPIs, H2RAs, statins, immunosuppressants and transfusion of red blood cells.

^c^Reference category: no mirtazapine and no trazodone.

## Discussion

Our findings suggest that depression and/or the use of specific anti-depressants are associated with development of CDI. Data from this nationally representative sample of older Americans show that population-based rates (which include both community-acquired and hospital-acquired disease) are greater in individuals with major depression and depressive disorders, and in people who reported feeling sad or having emotional, nervous, or psychiatric problems, after adjustment for confounders. In addition, widowed individuals and those living alone were significantly more likely to develop CDI. Results from our clinical study indicate that there may be certain anti-depressants that impart an altered risk for CDI, particularly the combination of mirtazapine with trazodone. Such effects were independent of antibiotic use.

There is experimental and epidemiologic evidence to support the hypothesis that depression and bereavement result in changes in the gastrointestinal (GI) system [7-10,15-17]. In a mouse model, depression (that is, maternal separation) was shown to result in both the development and greater severity of colitis [10]. In humans, pyrosequencing analysis of fecal microbiota of depressed patients showed distinct bacterial phylotypes compared with those found in patients without depression [16]. Moreover, patients with depressive symptoms exhibit greater and more prolonged inflammatory responses after antigen challenge than individuals without depressive symptoms, suggesting that depression may result in immune dysregulation [18]. Bereavement has also been shown to decrease production of neutrophil superoxides in older adults [19] and to reduce the functional activity of natural killer cells [20]. It is possible that the physiologic sequelae of depression itself, and not anti-depressants *per se*, may be associated with CDI risk.

It has been suggested that the mechanisms underlying the brain–gut axis may be bidirectional [21-23]. In a 12-year prospective study, the relationship between anxiety, depression, and functional GI disorders appeared to be bidirectional, in that psychiatric disorders predicted GI disease and *vice versa*[21]. A population-based longitudinal study in the Netherlands found similar results; the risk of developing severe bowel disease was significantly higher in individuals with previous depression, and the risk of developing depression was significantly higher in individuals who had previously experienced severe bowel disease [22]. The authors concluded that depression and severe non-cancerous bowel disease were ‘varying expressions of a partly shared aetiological process.’ [23] Animal studies have suggested similar effects, with the possibility that this interplay may begin in early life [23,24]. Our studies suggest that depression, widowhood, living alone, and the use of antidepressant medications preceded the onset of CDI. We found, in our nationally representative sample, that 35% of those who developed CDI were diagnosed with major depression before the infection. However, we did not have the complete medical history of the subjects prior to study entry and therefore, cannot be certain whether CDI pre-dated study entry for some of the subjects. It is possible that there is a lifelong liaison between the gut microbiota and neurologic response to external stimuli.

In our population-based Study 1, individuals with major depression had rates of *C. difficile* infection that were consistently elevated across all age groups (Table 1). For people without major depression, the rates of CDI increased with age. Note that the rate of CDI was 332.9/100,000 person-years in the youngest age group of patients with depression, whereas it was 301.0/100,000 person-years in the oldest age group for those without depression. If CDI were a crude indicator of gut health, it would seem that the microbiota of people with depression may be more similar to that of the very aged. Studies of the microbiota of older adults generally show less diversity, particularly in those who live in long-term residential care compared with community dwellers [25].

The relationship between depression and inflammatory bowel disease is well known [11,12,26]. A strong association between anti-depressants and inflammatory pouch syndrome has also been shown (adjusted OR of 4.17; *P* = 0.0002) [27]. Because of this, we adjusted for Crohn’s disease and ulcerative colitis in our studies, but the relationship between depression and CDI remained. This may indicate that mechanisms underlying colitis due to CDI and colitis due to inflammatory bowel disease share commonality, both involving host immune response and gut microbiota. When considered independently, each of these types of colitis was associated with depression.

We cannot completely discern whether it is the pathophysiology of depression itself or the treatment for depression that is the major driver of these findings. In the case–control study, we categorized the anti-depressants by their mechanism of action, but this categorization did not perfectly discriminate between the drugs that were associated with higher CDI risk from those that were not. It is widely appreciated that the mechanisms of action of many anti-depressants are complex, and all their effects (both intended effects and side-effects) are not fully characterized. Many of the anti-depressants work by altering serotonin levels and, in an animal model, Ghia and colleagues found that serotonin plays a key role in the pathogenesis of colitis [28]. However, even anti-depressants within the same general class may have slightly different actions; for example, fluoxetine has a longer half-life than other SSRIs, and has a greater incidence of weight loss and stimulant effects [29]. It is also possible that inter-patient variation in genes encoding for serotonin receptors may play a role in mediating responses to therapy. For example, in a double-blind randomized controlled trial of older patients taking mirtazapine versus those taking paroxetine, side-effects were strongly associated with the HTR2A C/C genotype in those receiving paroxetine but there was no effect with mirtazapine, suggesting that underlying genetic markers can also affect the mechanism of action [30]. In our investigation, the case-crossover design provided some insights regarding discrimination between effects due to the pathophysiology of depression itself versus those due to specific anti-depressants. In this study, personal history of depression was, by design, held constant because of the within-person comparison and therefore, any difference found in antidepressant use was not due to a history of depression. In effect, we measured drug use at different times (hospitalizations) in the same individual. In these analyses, we found that usage of most types of anti-depressants did not affect the likelihood of developing CDI. However, in a small group of patients, there was a significantly higher risk of CDI when taking both mirtazapine and trazodone together. The use of trazodone alone appeared protective, but when combined with mirtazapine, the risk was increased. Larger studies are necessary for confirmation of these findings. However, this only occurred in a minority of patients with CDI, which suggests that, for most patients, there are underlying pathophysiologic mechanisms of depression that result in their increased risk of CDI, independent of antidepressant use.

Whereas antibiotic use was closely associated with CDI, it was not evident that the link between depression and CDI was due to increased physician prescriptions for antibiotics in individuals with depression. In Study 2, we had a tightly matched group of controls to cases with respect to antibiotic use. All subjects were individuals whose stool samples were tested for *C. difficile,* and these tests were ordered when antibiotic-associated diarrhea was evident. We found that 83.8% of the cases and 82.6% of the controls had received antibiotics (*P* = 0.530). Furthermore, 82.8% of those receiving anti-depressants and 82.7% of those not receiving anti-depressants had been given antibiotics (*P* = 0.957). Adjustment for antibiotic use in the regression models (and for the number of doses of antibiotics) did not change the results.

Our investigation was limited by its observational design, especially for the assessment of anti-depressants, because effects of medication are best addressed through trials. However, CDI is not a common outcome, and a trial of such unintended effects may not be feasible. Although the relationship between anti-depressants and CDI was first reported in a study conducted in two hospitals [5], the types of anti-depressants in that study were not listed and therefore, we cannot adequately assess the similarity of results across hospitals. In Study 1, the use of a nationally representative sample that captured both outpatient visits and inpatient stays over an extended period (17 years maximum) was a strength for the assessment of depression, widowhood, and other factors in which randomization is not possible.

A limitation of Study 1 was the use of physician diagnoses to detect depression, which may underestimate the true frequency of this disease. However, data from the HRS contain additional information beyond physician diagnoses. Information was available from self-report by participants during biennial interviews. The variables shown in Table 2 relate to depression measured in different ways and the ORs all tended to show a similar pattern. For example, patient self-report of feeling sad (irrespective of a physician’s diagnosis) was significantly associated with CDI. The OR was 1.41 (*P* = 0.008) which parallels the findings for a physician diagnosis of major depression (OR = 1.36, *P* = 0.016). Moreover, if underestimation of depression is present through missed diagnoses, it is likely to be non-differential misclassification; that is, the under-reporting of depression could occur in both people who have and people who do not have CDI. There is currently no established link between depression and CDI in the medical literature by which physicians across the country would link these two conditions. When such non-differential misclassification occurs, the OR tends to be pulled towards the null. Thus, if under-diagnosis of depression does occur, we would expect that the true (reference population) OR would be greater than 1.36.

Another concern may be testing bias, which would occur if the ordering of a test for *C. difficile* occurs at different rates in persons with and without depression. In Study 2, participants were all patients who were tested for *C. difficile* during a given time period because of symptoms evidenced during hospitalization; that is, antibiotic-associated diarrhea. Because depression has not been previously correlated with CDI in the medical literature or in general clinical practice, we do not suspect that there was differential testing based on this specific diagnosis.

Unfortunately, questions regarding dietary intake were not asked of all participants in the HRS as a part of the ongoing biennial interview, nor did we have dietary intake information on the hospitalized patients in Study 2. Therefore, the influence of habitual diet on both depression and CDI cannot be assessed in our studies. There was one aspect of our investigation, however, in which we were able to control for past dietary intake. In the sensitivity study of the hospitalized patients (Study 2), we conducted a case-crossover study whereby each person was compared with him/herself. In this instance, past dietary habits (prior to July 2009) were held constant; such habits are the same because the person is the same. When diet was held constant, patients receiving mirtazapine with trazodone were at greater risk of developing CDI. However, if some of these patients began eating differently after July 2009, such differences could not be measured. Overall, the effect of diet on both depression and CDI would be an interesting area for further study.

## Conclusions

Given the rise of CDI, especially among older individuals [31], elucidating the modifiable risk factors for this often-fatal illness is an important patient safety issue. Our complementary studies reveal that adults with depression and those that use specific anti-depressants seem to be more likely to experience CDI. Widowhood and living alone are also associated with CDI. Clinicians prescribing antimicrobials to patients with depression should be aware of the possible increased risk of CDI in this patient population.

## Abbreviations

BMI: body mass index; CDI: *Clostridium difficile* infection; CI: Confidence interval; CMS: Centers for Medicare and Medicaid Services; GI: Gastrointestinal; HRS: Health and Retirement Study; H2RAs: histamine-2 receptor antagonists; PPIs: proton-pump inhibitors; SSRI: selective serotonin reuptake inhibitor; UMHS: University of Michigan Health System.

## Competing interests

The authors declare that they have no competing interests.

## Authors’ contributions

MAMR, MTG and DMA participated in designing the studies, and MAMR generated the hypotheses. VBY, KML and DMA provided resources. MAMR and MTG extracted the data. MAMR had full access to all of the data, conducted the analyses, and wrote the first draft of the report. MAMR, MTG, VBY, SS, KML, JYK and DMA participated in interpretation of the data, writing of the manuscript, and critically review of the paper. All authors approved the final manuscript.

## Authors’ information

The authors are part of the National Institutes of Health-sponsored Enterics Research Investigational Network Cooperative Research Centers that were funded to conduct research to bridge the gaps between basic, translational, and clinical research on enteric disease agents. As a part of this network, we are exploring contributors to *C. difficile* pathogenesis.

## Pre-publication history

The pre-publication history for this paper can be accessed here:

http://www.biomedcentral.com/1741-7015/11/121/prepub
